# Sequential Exploration Unearths Social Media to Influence the Persona of Practicing Pediatric Dentists

**DOI:** 10.21142/2523-2754-1303-2025-253

**Published:** 2025-08-31

**Authors:** Deepika Chari, Anup Panda

**Affiliations:** 1 PhD Scholar, College of Dental Sciences and Research Centre, Gujarat University, Ahmedabad, India. charideepika@gmail.com Ahmedabad India charideepika@gmail.com; 2 Professor & PhD Guide, College of Dental Sciences and Research Centre, Gujarat University, Ahmedabad, India. dranuppanda76@gmail.com Ahmedabad India dranuppanda76@gmail.com

**Keywords:** pediatric dentistry, social media, personality, questionnaire design, odontopediatría, redes sociales, personalidad, diseño de cuestionarios

## Abstract

**Background:**

The pediatric dentist's personality is subject to change owing to cultural, social, and technological changes. To achieve a successful treatment delivery, it is crucial to understand which aspect of a pedodontist's personality influences their clinical practice.

**Aim:**

This study aimed to explore the most influential factor that affects a pediatric dentist's personality. Methodology: This study used the mixed-methods sequential exploration technique. The qualitative phase included a semistructured interview (n=13) based on the Theoretical Domains Framework (TDF) and Capability-Opportunity-Motivation-Behavior (COM-B) model. Weightage analysis identified social media as the most common theme among the responses. A quantitative phase then followed wherein responses from a validated questionnaire (n=343) based on the technology acceptance model tested whether social media influenced a pediatric dentist's personality.

**Results:**

The inter-rater reliability using Krippendorff's Alpha in the qualitative phase was 0.82, indicating a strong agreement for theme identification (social media). The responses were grouped based on the participants' age: Group I (n=64; age <30 years), Group II (n=186; age 31-50), Group III (n=93; age >51 years). Intergroup comparison using the Kruskal-Wallis test showed statistically significant differences across questions related to perceived usefulness (n=3), perceived ease of use (n=3), attitude towards use (n=2), behavioral intention to use (n=1), and actual use (n=2). Post hoc analysis, conducted using Mann-Whitney U-tests, revealed significant variations in responses between Group 1 & Group 2, and between Group 1 & Group 3.

**Conclusion:**

Across various age groups, social media does influence a pediatric dentist's personality and is a key aspect of E-Professionalism.

## RESUMEN

**Antecedentes:**

La personalidad del odontopediatra está sujeta a cambios debido a los cambios culturales, sociales y tecnológicos. Para lograr una prestación de tratamiento exitosa, es crucial comprender qué aspecto de la personalidad de un odontopediatra influye en su práctica clínica.

**Objetivo:**

Este estudio tuvo como objetivo explorar el factor más influyente que afecta la personalidad de un odontopediatra. Metodología: Este estudio utilizó la técnica de exploración secuencial de métodos mixtos. La fase cualitativa incluyó una entrevista semiestructurada (n = 13) basada en el Marco de Dominios Teóricos (TDF) y el modelo Capacidad-Oportunidad-Motivación-Comportamiento (COM-B). El análisis de ponderación identificó las redes sociales como el tema más común entre las respuestas. Luego siguió una fase cuantitativa en la que las respuestas de un cuestionario validado (n = 343) basado en el modelo de aceptación de la tecnología probaron si las redes sociales influyeron en la personalidad de un odontopediatra.

**Resultados:**

La confiabilidad interevaluador utilizando Alpha de Krippendorff en la fase cualitativa fue de 0,82, lo que indica un fuerte acuerdo para la identificación del tema (redes sociales). Las respuestas se agruparon según la edad de los participantes: Grupo I (n=64; <30 años), Grupo II (n=186; 31-50 años), Grupo III (n=93; >51 años). La comparación intergrupal mediante la prueba de Kruskal-Wallis mostró diferencias estadísticamente significativas en las preguntas relacionadas con la utilidad percibida (n=3), la facilidad de uso percibida (n=3), la actitud hacia el uso (n=2), la intención de uso (n=1) y el uso real (n=2). El análisis post hoc, realizado mediante la prueba U de Mann-Whitney, reveló variaciones significativas en las respuestas entre los Grupos 1 y 2, y entre los Grupos 1 y 3.

**Conclusión:**

En diferentes grupos de edad, las redes sociales influyen en la personalidad del odontopediatra y son un aspecto clave del e-profesionalismo.

## INTRODUCTION

Personality, professionalism, and psychology are the 3Ps that interlink the work ethic of an individual. The latter is a broad field of study and a core human trait influencing the other two. This manuscript concerns the profession of a pediatric dentist, who routinely must undergo the delicate task of ensuring successful treatment delivery of children while ensuring that they are in control of their faculties.

The experiences of a pediatric dentist strongly influence their psychological traits and are thus reflected in their personality. The eventual persona of the pedodontist that surfaces has a direct implication on how the planned treatment is delivered.

As in every profession, the personality of a pediatric dentist is subject to change. Personality can be comprehensively understood through analogies of psychological theories as applied to pedodontics. Albert Bandura's theory suggests that, given the trends in technological and material advancements, pediatric dentists are likely to adapt by following peers through digital networking, thereby adopting a learning approach. ^1^

Other professional factors that mold a pediatric dentist's personality are the immediate feedback that a pediatric dentist receives from the patient's behavior and the overindulgence of parents. Icek Ajzen's Theory of Planned Behavior exemplifies that, for ethical practitioners, there is a nagging ability to resort to strategies that might not necessarily be evidence-backed. ^2^

Ambient factors like a supportive work environment and team enhance professional growth, which satisfies the social needs as theorized by Bandura. Most notable of these factors that can exponentially affect the personality of the pediatric dentist is Lewin's Theory of Change, which centers around the dogma of learning, unlearning, and relearning. Pediatric dentists can achieve this by attending workshops or continuing dental education programs and staying updated with the latest technological advancements that promote positive treatment delivery. ^3^^)^

The axioms mentioned above provide a framework for shaping the personality of pediatric dentists to ensure their profession remains relevant. Thus, this explorational study aimed to evaluate the most dominant factor that influences pediatric dentists of the current era.

## MATERIALS AND METHODS

After obtaining ethical clearance from the Institutional Ethics Committee of the College of Dental Sciences and Research Centre, Gujarat University, Ahmedabad, India (CDSRC/IEC/2023/26) to conduct this study from the institutional ethics board, an exploratory study design following the mixed-methods framework was designed for this study (see Figure 1). The study was also registered in the Clinical Trial Registry of India (CTRI/2023/08/056114). The study saw two phases: a) a qualitative phase and b) a quantitative phase. 

### Qualitative phase

This phase included semistructured interviews with twenty participants chosen through quota sampling. Each participant should have had a minimum of five years of professional experience and be well-versed with recent advances in the field of pediatric dentistry. Seven participants were eliminated for lack of satisfactory answers (as referred to by three different interviewers). Based on the recommendations of Guest et al. (2006) for a non-probabilistic sample for interviews without reaching data saturation, the eventual sample of 13 is justified.

The Theoretical Domain Framework (TDF) and Capability-Opportunity-Motivation-Behavior (COM-B) model were the basis for interview questions (Table 1). The semistructured interview conducted among the 13 pediatric dentists lasted for less than 15 minutes for each participant. It was either in-person or virtual, depending on feasibility and preferences. All interviewers focused on immediate and instinctive responses. If participants provided additional remarks, they were noted as valuable insights.

The interview transcripts of each participant were coded line-by-line. A weighted analysis deduced the factor that was the most common overarching element from the responses of each participant. An inter-rater reliability analysis ascertained that all three interviewers agree on the most prominent aspect influencing the personality of the participants.

**Table 1:** Interview questions based on the Theoretical Domain Framework (TDF) and Capability-Opportunity-Motivation- Behavior (COM-B) model

	Interview Questions	TDF	COM-B
1.	Do you think having an updated knowledge of recent advances influences your personality as a pediatric dentist during procedures?	Knowledge	Capability (Psychological)
2.	Do you think the ability to use recent equipment or perform updated techniques influences your personality as a pediatric dentist during procedures?	Skills	Capability (Psychological)
3.	Do you think all updated techniques or recent advances align for every case, and do they influence your personality as a pediatric dentist during procedures?	Beliefs about capabilities	Motivation
4.	Do you think the parents' behavior in the operatory influences your personality as a pediatric dentist during the procedure?	Intentions	Motivation (Reflective)
5.	When using a behavior guidance technique, do you need to adjust your approach based on the parent's behavior, and does this impact your personality as a pediatric dentist during the procedure?	Behavior regulation	Capability (Physical)
6.	As a follower of Evidence-Based Practice, when you execute your treatment plan, does the reliance on Evidence-Based Practice influence your personality as a pediatric dentist during the procedure?	Optimism	Motivation (Automatic)
7.	Does your personality change based on whether or not a goal was achieved (backed by Evidence-Based Dentistry)?	Goals	Motivation (Reflective)
8.	Does your personality change when there is varied evidence for a treatment plan?	Memory, attention, & decision	Capability (Psychological)
9.	By attending conferences/ workshops regularly to refine your clinical skills, does it affect your personality?	Reinforcement	Motivation (Automatic)
10.	Does pre- or during-training or counseling of the patient/parent influence your personality as a pediatric dentist during the procedure?	Beliefs about consequences	Motivation (Reflective)
11.	Does the reciprocation of the patient after behavior training drive you emotionally, which influences your personality as a pediatric dentist during the procedure?	Emotion	Motivation (Automatic)
12.	When you think of your behavior in the social domain, does it influence your personality as a pediatric dentist during a procedure?	Social or professional role & identity	Motivation (Reflective)
13.	Does the dental operatory (clinical ambiance, personnel) influence your personality as a pediatric dentist during the procedure?	Environmental context	Opportunity (Physical)
14.	Does your activity on social media, the parents' knowledge via social media, and the clinical updates by peers on social media influence your personality as a pediatric dentist during the procedure?	Social influences	Opportunity (Social)

### Quantitative phase

Since the most commonly identified factor that emerged in the qualitative phase was social media, a customized questionnaire was formulated based on the Technology Acceptance Model (TAM). The null hypothesis was that social media does not affect a pediatric dentist's personality. An expert group consisting of four pediatric dentists made a customized, validated questionnaire to test this. After three rounds of Delphi consensus, researchers formulated 30 questions based on the Target-Action-Context-Time principle.

This questionnaire was pilot-tested on 33 pediatric dentists. Based on their responses, an analysis of internal consistency eliminated eight questions. The final questionnaire consisted of 22 questions. The TAM model helped in anchoring these questions as follows: perceived usefulness (5 questions), perceived ease of use (5 questions), attitude towards use (4 questions), behavioral intention to use (4 questions), and actual use (4 questions). Each question was to be answered on a 5-point Likert scale (1 - Strongly Disagree, 2 - Disagree, 3 - Neutral, 4 - Agree, 5 - Strongly Agree) (see Table 2).

**Table 2:** Interview questionnaire based on the Technology Acceptance Model (TAM). Each component is rated on a Likert scale (1: Strongly Disagree, 2: Disagree, 3: Neutral, 4: Agree, 5: Strongly Agree)

Components of TAM	Interview Questions
Perceived Usefulness (U)	1. Social media would help me improve my professional growth and knowledge 2. Social media would help me to communicate clearly with my patients and other colleagues 3. Social media would help me promote my dental practice 4. Social media would help me stay updated with the latest trends in pediatric dentistry 5. Social media would help me make clinical decisions.
Perceived Ease of Use (E)	6. I find it easy to use various social media platforms for professional purposes. 7. I find it easy to create relevant (dental-related) content regularly 8. I find it easy to connect with my patients and their families through social media 9. I find it easy to maintain my professional presence on social media 10. I find it easy to learn the technical aspects to navigate social media
Attitude towards use (AU)	11. I feel comfortable using social media for professional purposes 12. I believe the usage of social media will add value to my professional growth 13. I feel confident that social media will boost my professional image 14. I believe spending time on social media for professional purposes is worthwhile
Behavioral intention to use (BI)	15. I intend to learn new methods to improve my usage of social media for professional purposes. 16. I intend to remain consistent in preparing social media content related to pediatric dentistry. 17. I intend to recommend social media to other dentists for their professional growth. 18. I intend to expand my social media reach among existing patients and the general public.
Actual use (A)	19. Social media has helped me increase my patient flow 20. Social media has helped me increase my presence in the professional community 21. Social media has helped me prepare and display content that could increase awareness and educate the general population 22. Social media has helped me improve my ability to network with other dental professionals

At the time of finalizing the questionnaire, the Indian Society of Pedodontics and Preventive Dentistry (ISPPD) had 2633 members. Five hundred of these members (~20% of the population of interest) received the questionnaire through a Google Form. The responses of participants were tabulated based on the age groups of the participants. These responses identified three groups: Group I (n=64; age <30 years), Group II (n=186; age 31-50), Group III (n=93; age >51 years). Later, R software (version 4.3.3) statistically analyzed the data.

## RESULTS

Weighted analysis of the themes identified by line-by-line coding of the interview transcripts revealed social media to be the most influential factor (see Table 3). The inter-rater reliability in the qualitative phase was 0.82 as per Krippendorff's Alpha, indicating a strong agreement for theme identification (social media).

**Table 3:** Weighted Analysis

THEMES	FREQUENCY	POINTS	SCORE
Social media	17	9	153
Child's behavior	12	8	96
Confidence	11	7	77
Execution	11	6	66
Exposure	10	5	50
Patient's best interest	9	4	36
Training	8	3	24
Conventional	8	2	16
Uncooperative parents	7	1	7
Working space	7	-	-
Dental assistant	7	-	-
Studies	6	-	-
Inconsistent knowledge translation	6	-	-
Consultant	6	-	-
PubMed	5	-	-
Parents' feedback	5	-	-
Parents' consent	4	-	-

The 343 responses received in the quantitative phase signified a high response rate (68.6%). Intergroup comparison using the Kruskal-Wallis test showed that responses to 11 of the 22 questions were significantly different across the three age groups (see Table 4). These questions belonged to perceived usefulness (n=3), perceived ease of use (n=3), attitude towards use (n=2), behavioral intention to use (n=1), and actual use (n=2). Questions 13 (H = 10.22, p = 0.006) and question 5 (H = 9.96, p = 0.006) showed the most significant difference.

**Table 4:** Statistical Analysis

Question Number	TAM	H-Statistic	p-value
2	U	6.77	0.033
3		8.19	0.016
5		9.96	0.006
6	E	7.82	0.02
7		7.79	0.02
8		6.07	0.047
12	AU	6.9	0.031
13		10.22	0.006
15	BI	7.8	0.02
19	A	6.92	0.031
20		8.87	0.011

Post hoc analysis using Mann-Whitney U-tests revealed significant variations in responses to 12 questions between Group 1 and Group 2, and four questions between Group 1 and Group 3. No significant observations were recorded in the pairwise comparison of Group 2 vs Group 3. The effect sizes observed were G1 vs G2 (r = 0.33), G2 vs G3 (r = 0.32), and G1 vs G3 (r = 0.30), indicating a moderate difference between groups (see Table 5).

**Table 5:** Statistical Analysis (Post-hoc analysis)

Comparison	Question number	TAM	U-Statistic	p-value	Effect size
G1 vs G2	2	U	1.5	0.027	0.33
	3		0	0.007	
	5		0	0.007	
	6	E	0	0.007	
	7		1	0.021	
	8		1	0.015	
	9		2	0.031	
	12	AU	1	0.015	
	13		0	0.007	
	15	BI	1	0.02	
	19	A	0	0.011	
	20		0	0.011	
G2 vs G3	No statistical significance observed				0.32
G1 vs G3	3	U	23	0.036	0.3
	5		24.5	0.015	
	13	AU	25	0.007	
	20	A	22.5	0.046	

## DISCUSSION

Personality traits have a greater influence in a profession where decision-making, and patient communication with parent satisfaction are essential. Empathy, patience, and adaptability are among the few traits that are crucial for any pediatric dentist. ^4^

A stepwise exploration method facilitated a deep understanding of the key factors influencing the personality traits of pediatric dentists and their impact on practice management. ^5^^)^ The inclusion of semistructured interviews in this study allowed participants to provide open-ended answers, ensuring consistency in responses. ^6^

The first step of the stepwise exploration method qualitatively derived the factors that can influence the behavior of the pediatric dentist, which in turn, are reflected in their dental practice management. We achieved this by formulating interview questions based on the Theoretical Domains Framework - Capability, Opportunity, Motivation (TDF-COM-B) behavior model using the Target Action Context Time (TACT) principle. ^7^

Thematic coding and weightage analysis revealed the phrase 'social media' (and its variants) as the most contributing factor in behavior change. A previous cross-sectional study has explored the effect of social media on personality change. ^8^^)^ Furthermore, the cultural evolutionary theory states that social change over time becomes the basis of an individual's changing behavior, which also impacts their profession.

The second step of stepwise exploration was to test the insights derived from the first step, and thus, a cross-sectional study was structured based on the Technology Acceptance Model (TAM). The syntax of TAM facilitates understanding of the attitudes, intentions, and usage of social media by pediatric dentists and how it affects them in professional contexts. There is evidence of published literature suggesting the use of the TAM model to explore the impact of social media on healthcare professionals. ^8^

The second generation of the World Wide Web began in 2004 (Web 2.0). Four years later, dental-related blogs started. This chronological evolution hints that a range of dental professionals from various age groups have encountered this transition, wherein they used social media on the professional front. ^9^^)^ Thus, participants were grouped in this study based on age groups.^10^^)^ A noteworthy comparison between the variations in responses of budding professionals and veterans is a macroscopic observation of the results of our study. Notably, the responses by the participants between 31 and 50 years of age merit a separate discussion, given the heterogeneity in their responses that do narrate a story.

Interactive learning and networking reached their peak in the Web 2.0 era. Features such as mass participation, user participation, and folksonomy have given dentists a positive edge not only in personal branding but also in professional identity enhancement. ^11^ The results of our study showed that participants from the youngest age group preferred social media for their presence rather than communicating with the patients. It implied that they create content, including blogs, vlogs, and posts, instead of direct patient communication. ^9^^)^ The results of our study echo the findings of a previous survey among US dentists, as personal branding is a form of marketing. They showed that marketing dental practice and maintaining an online presence were the most common uses of social media. ^12^^)^ Another interesting response saw that the older age group believed that taking help for clinical decisions was a righteous use of social media. This finding aligns with the observations of Craig M (2019), who stated that participants from the centennial and millennial generations preferred to get involved in 'crowd sourcing' if needed to take expert opinions on complex procedures. ^13^

The altruistic behavior of the veterans, combined with their ethical knowledge, is reflected in our study, which showed that G3 participants primarily used social media for professional purposes. ^14^^)^ Being familiar with evidence-based dentistry, they might try to avoid being influenced by 'Instagram dentistry.' This approach makes their content more dental-related while minimizing the potential pitfalls of social media platforms. When drafting such content, conscious efforts are needed to consider the emerging phenomenon of cyberchondria. ^13^^)^

Participants of G2 also believed in providing engaging content that could build up their online presence. They also showed affinity towards connecting with the patients through social media. However, it may involve sharing clinical data to enhance visibility, reach, and stay relevant in their professional fraternity. Such an act is characteristic of ethical egoism rather than utilitarian behavior.

The transition from interpersonal to professional interactions has the potential to create a conundrum in the relationship status of dentists with their patients. It might lead to a breach of E-Professionalism, where apart from sharing dental-related posts, they indulge in 'liking' and 'commenting' on personal posts. ^15^ The Health Insurance Portability and Accountability Act (HIPAA) states that a dentist should have separate accounts for personal and professional use. They should refrain from 'friending' their patients. By doing so, they ensure they maintain the fundamental standards of privacy, confidentiality, and security. ^8^

In the competitive Web 2.0 era, pediatric dentists utilized social media to boost their professional image by showcasing their cases or achievements. Such a move is seemingly lucrative to reach the masses, especially when it is quick and cost-effective.^16^^)^ This aligns with our finding that the younger generation aims to reap 'impressions', neglecting the consequences of leaving digital footprints.^17^^-^^19^^)^ As stated earlier, the participants of G2 believe that social media will help them boost their professional presence; however, their skewed responses show signs of skepticism on whether this digital mode will maximize their professional growth.

The younger generation of professionals is exposed to the digital prowess of social media early in their careers. Being tech-savvy, the G1 participants expressed behavioral intention to learn new social media operations that can help them engage actively in dental discussions and enhance their dental practice. This character encourages them to involve the 'fourth parties' like web designers, social media promoters, paid reviewers, and coupon brokers. ^10^^,^^16^^)^ The involvement of fourth parties might end up in the 'Groupon effect'. 'Patient poaching' promotes this practice. It involves social couponing or enticing that might initially seem to have an explosive effect of attracting patients. Still, it later sees a steep decline, indirectly damaging the essence of a noble profession. ^20^

There exist cultural disparities in the selection of dentists by potential patients who browse social media to search for a credible dentist. The sharing of patients' photographs (pre- and post-operative images) on social media is an influential trend, with a predilection among females.^17^^)^ In another cross-sectional study, it was shown that irrespective of the patient's relationship with the dentist, how patients’ photos are shared directly corresponds to the professional credibility of an orthodontist.^21^^)^ Intergroup comparison from our study revealed that participants from G2 opted to grow their presence in the professional community through sharing clinical cases on social media.

The effectiveness of social media as a tool to promote patient flow awaits a stringent study design. Two recent studies that explored this subject drew contrasting results. A 2023 Spanish web-based questionnaire study deduced that patients would change their dental practitioner whenever a dental practice promotes its facilities and technology. ^22^ On the other hand, a Serbian study with over 980 participants showed that social media was not an essential factor in choosing a dentist, with the most concerning factors being credibility and promotion of harmful trends. Furthermore, the participants of this study considered the age and education level of the dentist over any other factor in choosing a dentist. ^19^^)^ The results of the latter study draw parallels with the actual use of social media among participants of our study, wherein the veterans state that social media has helped them increase their patient flow.

This exploratory study warrants a discussion of its strengths. Firstly, the triangulation technique of mixed methods research helped in an effective deduction of a personality trait that influences the dentist of today. Furthermore, deducing an abstract emotion and testing how it affects a scientific profession is the highlight of our work. Finally, the cross-sectional survey's inferences allowed discussions of the various facets of E-professionalism in a technology-based study model. 

This study has three notable biases. The first is a universal limitation of any self-reported survey, which is acquiescence bias or social desirability bias. Next, since all the participants are from India, a geographical bias can be implied. Finally, we confess an interpretation bias from our end, wherein we were likely to avoid the objective measurement of the social media activity of the participants.

Social media is a double-edged sword that has the potential to provide both opportunities and challenges. On one hand, where a dentist can expand his profession by adapting to Instagram dentistry, Facebook intrusion (excessive use of Facebook that interferes with daily activities and interpersonal relationships) has become a significant concern that affects the dentist-patient relationship. ^23^

Our work offers an opportunity for future research that can offer a carefully curated social media ethics protocol. The rise of artificial intelligence and the need to keep up with technological advancements demand the inclusion of digital professionalism training in the dental curriculum. Finally, a probe on all the factors (apart from social media) in our weighted analysis can help us understand how they influence the personality of the pediatric dentist.

## CONCLUSION

As it is commonly said, age is just a number, and so is the relevance of age with the usage of social media. All three age groups were in some or other way influenced by social media, depending on their knowledge, availability, and requirements.
